# Dual-mechanism driven adsorption on silica microspheres enables precision pharmacokinetic profiling of liposomal therapeutics

**DOI:** 10.1016/j.jpha.2026.101714

**Published:** 2026-07-08

**Authors:** Jinjing Che, Yixuan Zhang, Mei Yuan, Qianqian Bai

**Affiliations:** Laboratory of Advanced Biotechnology, Academy of Military Medical Sciences, Beijing, 100850, China

## Abstract

• SiO_2_ microspheres accurately separated encapsulated, free, and total liposomal drug forms in plasma with cost efficiency.
• Silica hydroxyl and siloxane groups bind liposomal phospholipids via dual hydrogen bonding and electrostatic interactions.
• The method offers high sensitivity, precision, and recovery for pharmacokinetic analysis of the three liposomal drug forms.
• This validated approach may be developed into a universal method for liposomal drugs.

## Graphical abstract

**Figure:**
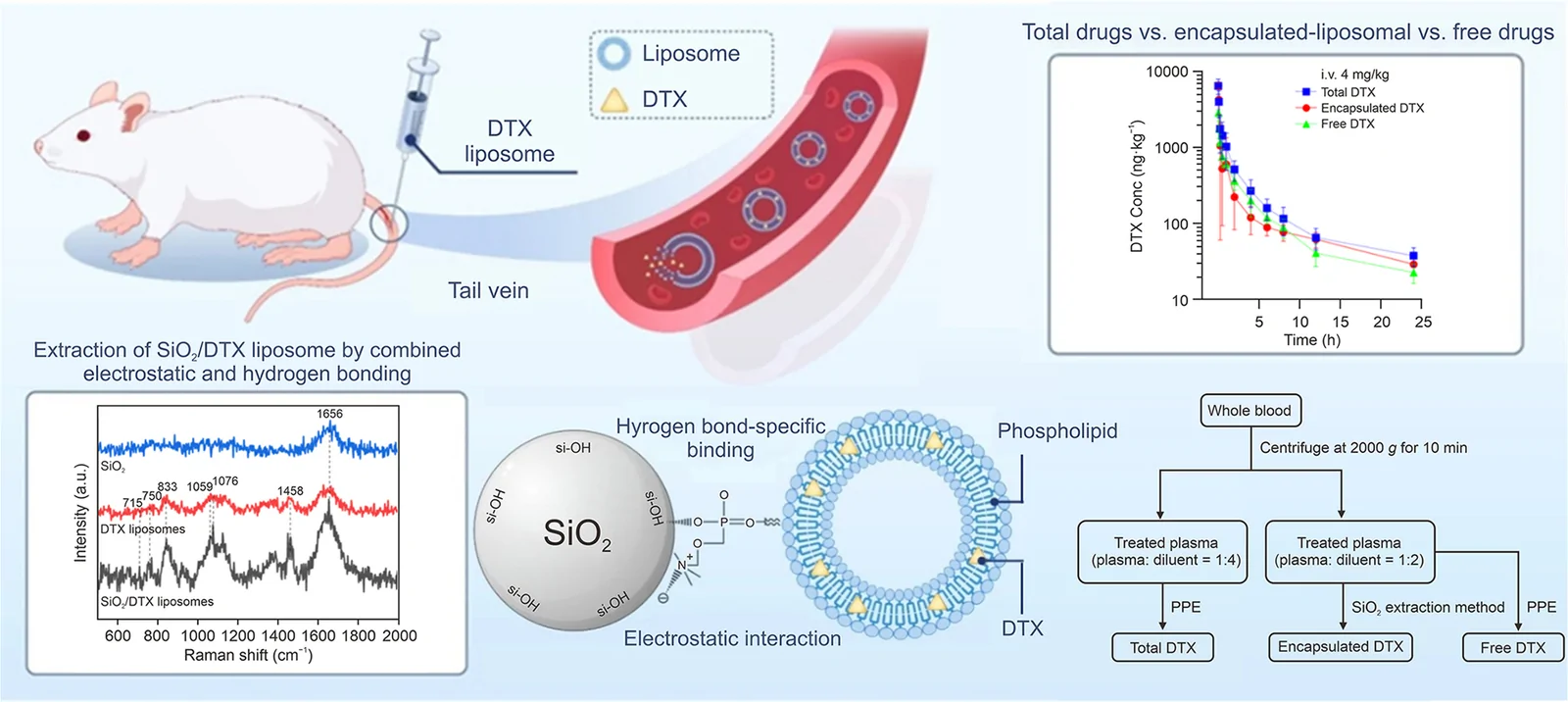

Since 1995, 15 liposomal drugs have been approved by the US Food and Drug Administration (FDA) and European Medicines Agency (EMA), enhancing drug solubility and targeting, as seen with docetaxel (DTX). Liposomal DTX formulations have entered clinical trials. Accurate pharmacokinetic analysis requires distinguishing between total, encapsulated, and free drug forms *in vivo*. Current separation methods like solid-phase extraction have issues such as adsorption and leakage. Previously, TiO_2_ microspheres were used for DTX liposome studies, but they are costly and complex [1]. Liu et al. [2] showed that SiO_2_ microparticles can adsorb phosphatidylcholine lipids to form stable lipid bilayers. We introduce a new method using SiO_2_ microspheres to separate free and encapsulated DTX in plasma. Transmission electron microscope (TEM) and flow cytometry analyzed DTX liposome interactions with SiO_2_ microsphere (Fig. S1), while Raman spectroscopy, isothermal titration calorimetry (ITC), and Langmuir modeling explored molecular mechanisms and adsorption kinetics. The optimized method offers a quick, simple, and cost-effective solution for pharmacokinetic profiling of DTX liposomes. The method may be extended to other liposomal formulations based on the interaction between SiO_2_ microspheres and phospholipid.

TEM images reveal that DTX-loaded liposomes, approximately 100 nm in diameter, maintain their structural integrity both before and after adsorption onto SiO_2_ microparticles, which are approximately 5 μm in size. Initially, SiO_2_ microparticles exhibit clean, uniform, and spherical surfaces. Following incubation, a dense layer of liposomes, each about 100 nm in diameter, adheres to the SiO_2_ surfaces without any observable structural damage. High-magnification TEM analysis further confirms the preservation of liposome integrity after adsorption (Fig. 1A).

**Fig. 1:**
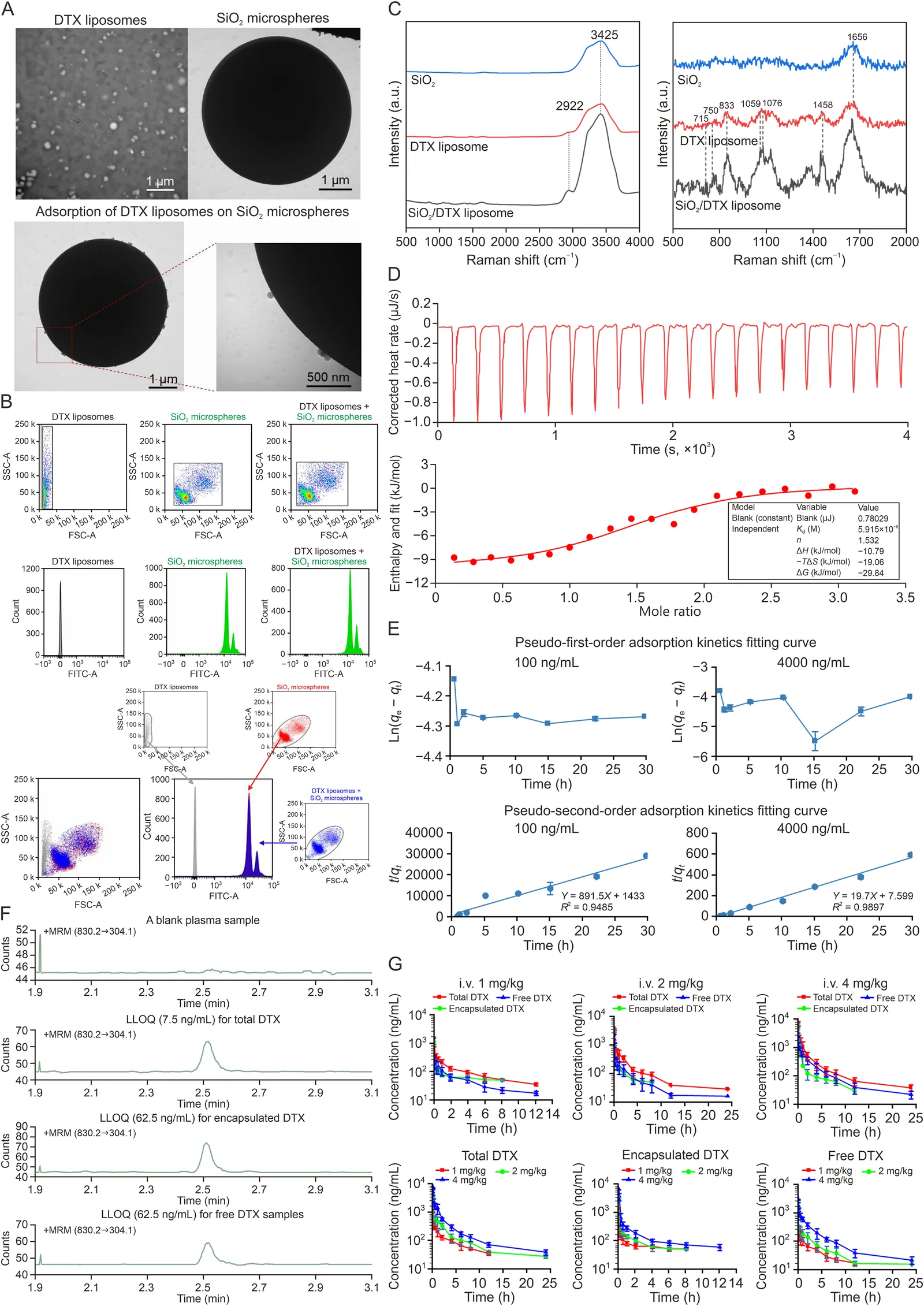
Dual-mechanism driven adsorption on silica microspheres enables precision pharmacokinetic profiling of liposomal therapeutics. (A) Transmission electron microscopy images. (B) Characterization of the adsorption of docetaxel (DTX) liposomes onto SiO_2_ microspheres by flow cytometry. (C) Raman spectroscopic analysis of SiO_2_ microspheres, DTX liposomes, and the SiO_2_/DTX liposome complex. (D) Isothermal titration calorimetry (ITC) analysis of the interaction between 500 μM DTX liposomes and 50 μM SiO_2_ microspheres at pH 5. (E) Adsorption kinetics of DTX liposomes onto SiO_2_ microspheres at different plasma drug concentrations. (F) Representative chromatograms of analytes in plasma samples. (G) Pharmacokinetic profiles of DTX liposomes after intravenous administration in rats. FSC: forward scatter; SSC: side scatter; FITC: fluorescein isothiocyanate; LLOQ: lower limit of quantification.

Flow cytometry analysis revealed that DTX liposomes adhere to fluorescein isothiocyanate (FITC)-labeled SiO_2_ microspheres without fusing or being damaged, as indicated by consistent forward and side scatter profiles before and after adsorption (Fig. 1B). The disappearance of the liposome signal and overlapping scatter distributions confirm complete surface adsorption rather than aggregation or internalization. Unchanged side scatter (SSC) signals suggest no significant surface roughness or molecular changes, supporting monolayer adsorption. Unlabeled DTX liposomes showed little FITC signal (1.1 k), and their peak disappeared with SiO_2_ addition, confirming full surface adsorption. The scattering profiles of the SiO_2_-liposome complex matched those of bare SiO_2_ microspheres, with no new optical features signals. Overlapping signals in flow cytometry dot plots and histogram confirm surface attachment of liposomes rather than fusion or aggregation. A slight increase in SSC indicates enhanced surface complexity due to liposome adsorption, while unchanged signal positions suggest no structural disruption.

Raman spectroscopy suggests that DTX liposomes adsorb onto SiO_2_ microspheres through a combination of electrostatic interaction and hydrogen bonding (Fig. 1C). The 3425 cm^−1^ peak shows stronger O–H bond stretching in SiO_2_/DTX liposome samples than in isolated silica microspheres or DTX liposome samples, likely due to hydrogen bonding between SiO_2_ microspheres and DTX liposomes. The 750 cm^−1^ peak associated with tetraalkylammonium groups in liposomes, splits into a doublet at 715/750 cm^−1^, suggesting electrostatic interactions between dissociated silanol groups on SiO_2_ and quaternary ammonium ions on the liposome [3]. Peaks at 833 cm^−1^ and 1458 cm^−1^ indicate C–H bond bending in the liposome's methyl groups. Increased peak intensity in SiO_2_/DTX liposome samples compared to liposome-only samples suggests lipid adsorption on SiO_2_ microspheres. The P=O group in phosphates (PO_4_^3−^) usually vibrates between 1000 and 1200 cm^−1^, and a blue shift from 1059 to 1076 cm^−1^ with increased intensity suggests hydrogen bonding between PO_4_^3−^ in liposomes and silanol on SiO_2_ microspheres. The O–H bending vibration peak at 1656 cm^−1^ appears in all samples, but the SiO_2_/DTX liposome sample exhibits much higher intensity, indicating stronger hydrogen bonding during adsorption. SiO_2_ has a strong affinity for tetramethylammonium ions (TMA^+^), unlike TiO_2_, which shows weak interaction even at high TMA^+^ concentrations [3]. This suggests that SiO_2_ microspheres are more efficient for liposome adsorption than TiO_2_ for liposome drug pharmacokinetics.

The isothermal titration calorimeter results suggest that liposomes exhibit strong binding affinity to SiO_2_ microspheres, characterized by a dissociation constant (*K*_d_) of 5.92 × 10^−6^ M (Fig. 1D). The negative enthalpy change (ΔH = −10.79 kJ/mol) and entropy change (ΔS = −63.93 J/mol/K) indicate that the adsorption process is driven by a combination of hydrogen bonding and electrostatic interactions, resulting in an exothermic and order-increasing event. The negative Gibbs free energy change (ΔG = −29.84 kJ/mol) further confirms the thermodynamic spontaneity of the process. Additionally, the binding ratio (n ≈ 1.532) suggests monolayer coverage.

The adsorption of docetaxel liposomes onto SiO_2_ microspheres follows pseudo-second-order kinetics [4], as indicated by a better linear correlation coefficient (*r*^2^) and closer agreement between theoretical and experimental *q*_e_ values (Fig. 1E). Optimal conditions include the SiO_2_ mass of 3 mg, 2-fold plasma dilution, pH 5, a 15-min incubation, and a particle size of 5 μm (Fig. S2).

Method validation for total, encapsulated, and free docetaxel was conducted to support pharmacokinetic studies (Fig. 1F, and Table S1–S6), adhering to regulatory guidelines [5]. The pharmacokinetic experimental protocol was approved by the Animal Ethics Committee of the Beijing Institute of Pharmacology and Toxicology (Animal Certificate Number: SCXK (Jing) 2021–0006; Ethics Approval Number: IACUC-DWZX-2024-P693), and all procedures involving animals complied with the Guidelines for the Care and Use of Laboratory Animals. In rat pharmacokinetics studies, intravenous docetaxel liposomes (1–4 mg/kg)showed linear AUC_0–*t*_ increases for all forms, confirming linear pharmacokinetics (Fig. 1G). All forms peaked at 2 min post-injection; encapsulated docetaxel had a longer *t*_1/2_ than free docetaxel, indicating prolonged circulation. The shorter *t*_1/2_ of free docetaxel and its slow release may be associated with a lower risk of acute toxicity. The markedly larger volume of distribution of encapsulated docetaxel is consistent with enhanced tissue uptake, which is often interpreted as a prerequisite for the enhanced permeability and retention (EPR) effect (Table S7). Unlike TiO_2_ extraction methods, the SiO_2_ approach provides a dependable and complete *in vivo* biodistribution profile.

This study presents SiO_2_ microsphere method for extracting encapsulated docetaxel liposomes and separating free docetaxel from plasma for pharmacokinetic analysis (Fig. S2). Liposomes appear to uniformly adsorb on SiO_2_ microspheres without detectable leakage, likely driven by a combination of hydrogen bonding and electrostatic interactions, which may offer improved specificity and efficiency over TiO_2_-based methods. The negative ΔG indicates spontaneous adsorption, and the binding ratio (∼1.532) aligns with Langmuir kinetics. Validation in rat plasma confirmed the method's accuracy and reliability. Pharmacokinetic study showed a linear dose-response for all docetaxel forms, with encapsulated docetaxel having a longer *t*_1/2_, lower *C*_max_ for free docetaxel, and larger *V*_ss_, suggesting extended circulation, reduced toxicity, and improved tissue distribution. This approach supports pharmacokinetic profiling of liposomal therapeutics and may be extended to other liposomal formulation based on the interaction between SiO_2_ microspheres and phospholipid.

## CRediT authorship contribution statement

**Jinjing Che:** Writing – review & editing, Methodology, Investigation, Conceptualization. **Yixuan Zhang:** Writing – original draft, Validation, Methodology. **Mei Yuan:** Validation. **Qianqian Bai:** Visualization.

## Declaration of competing interest

The authors declare that they have no known competing financial interests or personal relationships that could have appeared to influence the work reported in this paper.
