# Supplementary material for: Dual-mechanism driven adsorption on silica microspheres enables precision pharmacokinetic profiling of liposomal therapeutics
Source: J Pharm Anal. 2026 Jul 8;16(9):101714. doi: 10.1016/j.jpha.2026.101714 (PMC13602245; doi:10.1016/j.jpha.2026.101714)
Supplement: Supplementary file 1 [file mmc1.docx]

**Supplementary Material**

**Fig. S1.** Scheme of sample collection, extraction, and docetaxel (DTX) analysis methods. (A) Scheme of sample collection and extraction. (B) total DTX method: protein precipitation extraction (PPE). (C), encapsulated DTX method (SiO_2_ extraction method) and free DTX method (PPE). The lipid bilayer (non-modified) of DTX liposomes primarily consisted of semi-synthetic lipids, including distearoylphosphatidylcholine (DSPC), cholesterol, and a small amount of polyethylene glycol 2000-distearoylphosphatidylethanolamine (PEG2000-DSPE, approximately 2%). The encapsulation efficiency was 91.7%, with an active pharmaceutical ingredient (API) content of 7.5 mg per vial and a zeta potential of -6.45 mV. Docetaxel, with a purity of >98%.

**Fig. S2.** Optimization of the SiO_2_ conditions for liposome extraction. (A) Determination of the optimal mass of SiO₂. (B) Determination of the optimal dilution factor. (C) Determination of the optimal pH. (D) Determination of the optimal incubation time. (E) Evaluation of the particle size of SiO₂ microspheres (1 μm, 3 μm, and 5 μm). Non-modified SiO₂ microspheres with particle sizes of 1 μm, 3 μm, and 5 μm, as well as green fluorescent SiO₂ microspheres, were purchased from Tianjin Junyijia Technology Co., Ltd. These microspheres are negatively charged on their surface and are non-porous.

Table S1 Intra- and inter-run accuracy and precision of QC samples for three analytes in five assays.

| Analyte | Concentration  (ng·mL^−1^) | %CV | | %Bias | |
| --- | --- | --- | --- | --- | --- |
|  |  | Intra-day | Inter-day | Intra-day | Inter-day |
| Total DTX | LLOQ (7.5) | 6.41 | 5.67 | 0.77 | −0.05 |
|  | LQC (30) | 3.16 | 3.06 | −8.05 | −7.24 |
|  | MQC (300) | 4.08 | 3.47 | −1.00 | −1.01 |
|  | HQC (3000) | 3.70 | 3.27 | −2.39 | −3.25 |
| Encapsulated DTX | LLOQ (62.5) | 7.44 | 8.27 | 6.88 | 7.25 |
|  | LQC (100) | 3.06 | 2.08 | −13.7 | −13.4 |
|  | MQC (300) | 4.60 | 4.20 | −6.86 | −6.05 |
|  | HQC (3000) | 0.79 | 3.82 | 12.7 | 11.0 |
| Free DTX | LLOQ (15) | 6.20 | 5.61 | 0.64 | 0.55 |
|  | LQC (30) | 2.19 | 3.22 | 3.50 | 3.26 |
|  | MQC (300) | 2.18 | 3.41 | 2.24 | 2.35 |
|  | HQC (3000) | 2.85 | 4.11 | 4.98 | 2.64 |

* QC: quality control; CV: coefficient of variation; DTX: docetaxel; LLOQ: lower limit of quantification; LQC: low quality control; MQC: medium quality control; HQC: high quality control.

Table S2 Linearity of total DTX (n = 5).

| Total DTX Conc  (ng·mL^−1^) | Back-calculated concentrations of calibration standards for total DTX (Linear weighted 1/x^2^) | | | | | | | | |
| --- | --- | --- | --- | --- | --- | --- | --- | --- | --- |
| Run number | 7.5 | 25 | 50 | 125 | 250 | 500 | 1000 | 2000 | 4000 |
| 1 | 7.48 | 25.8 | 44.5 | 123.7 | 247.6 | 488.8 | 1086.0 | 2084.9 | 3937.4 |
| 2 | 7.54 | 26.4 | 50.6 | 130.0 | 259.9 | 493.9 | 987.2 | 2054.7 | 3487.6 |
| 3 | 7.62 | 26.3 | 44.1 | 128.2 | 288.8 | 493.4 | 982.3 | 1835.1 | 4010.5 |
| 4 | 7.15 | 26.7 | 47.4 | 141.0 | 263.9 | 474.2 | 941.0 | 1892.5 | 3944.0 |
| 5 | 7.49 | 25.8 | 44.5 | 123.7 | 247.6 | 488.8 | 1086.0 | 2084.9 | 3937.4 |
| Mean SD | 7.46 0.18 | 26.20 0.39 | 46.21 2.77 | 129.32 7.08 | 261.56 16.9 | 487.81 7.97 | 1061.5  66.0 | 1990.44 118.0 | 3862.4 212.4 |
| %CV | 2.41 | 1.48 | 6.00 | 5.48 | 6.50 | 1.63 | 6.49 | 5.93 | 5.50 |
| %Bias | −0.59 | 4.79 | −7.57 | 3.46 | 4.62 | −2.44 | 1.65 | −0.47 | −3.42 |

* DTX: docetaxel; SD: standard deviation; CV: coefficient of variation.

Table S3 Linearity of encapsulated DTX (n = 5).

| Encapsulated DTX Conc (ng·mL^−1^) | Back-calculated concentrations of calibration standards for encapsulated DTX (Linear weighted 1/x^2^) | | | | | | |
| --- | --- | --- | --- | --- | --- | --- | --- |
| Run number | 62.5 | 125 | 250 | 500 | 1000 | 2000 | 4000 |
| 1 | 66.55 | 112.83 | 258.56 | 460.37 | 951.63 | 2163.05 | 3397.24 |
| 2 | 67.24 | 122.80 | 239.91 | 459.55 | 974.09 | 2085.96 | 4375.88 |
| 3 | 64.61 | 120.93 | 239.43 | 477.73 | 979.83 | 2033.59 | 4364.10 |
| 4 | 64.65 | 127.15 | 242.91 | 497.68 | 1005.73 | 2028.74 | 4034.87 |
| 5 | 64.38 | 123.54 | 234.85 | 507.58 | 1002.07 | 2008.17 | 4130.48 |
| Mean  SD | 65.49 1.31 | 121.45 5.23 | 243.13 9.09 | 480.58 21.68 | 982.67 22.10 | 2063.90 62.41 | 4060.51 399.09 |
| %CV | 2.01 | 4.38 | 3.74 | 4.51 | 2.25 | 3.02 | 9.82 |
| %Bias | 4.78 | −2.84 | −2.75 | −3.88 | −1.73 | 3.20 | 1.51 |

* DTX: docetaxel; SD: standard deviation; CV: coefficient of variation.

Table S4 Linearity of free DTX (n = 5).

| Free DTX Conc (ng·mL^−1^) | Back-calculated concentrations of calibration standards for encapsulated DTX (Linear weighted 1/x^2^) | | | | | | | |
| --- | --- | --- | --- | --- | --- | --- | --- | --- |
| Run number | 15 | 50 | 125 | 250 | 500 | 1000 | 2000 | 4000 |
| 1 | 15.4 | 53.7 | 114.4 | 223.6 | 539.6 | 1043.3 | 2023.0 | 3961.1 |
| 2 | 15.5 | 52.0 | 112.5 | 252. | 544.1 | 1003.6 | 1970.1 | 3908.9 |
| 3 | 15.2 | 52.6 | 108.8 | 231.5 | 534.2 | 979.5 | 2116.5 | 4175.3 |
| 4 | 15.3 | 45.1 | 114.2 | 243.3 | 541.4 | 1069.6 | 2076.1 | 3933.7 |
| 5 | 15.3 | 48.8 | 113.8 | 237.7 | 537.1 | 1057.3 | 2041.6 | 3968.9 |
| Mean SD | 15.4 0.09 | 50.5  3.51 | 112.8 2.31 | 237.6  10.88 | 539.3 3.81 | 1030.7 37.86 | 2045.5 55.20 | 3989.6  106.51 |
| %CV | 0.61 | 6.95 | 2.05 | 4.58 | 0.71 | 3.67 | 2.70 | 2.67 |
| %Bias | 2.33 | 0.90 | −9.79 | −4.95 | 7.86 | 3.07 | 2.27 | −0.26 |

* DTX: docetaxel; SD: standard deviation; CV: coefficient of variation.

Table S5 Matrix effect and extraction recovery of liposome extraction for total, encapsulated, free DTX in rat plasma (n = 5).

| Analyte | Concentration (ng·mL^−1^) | Matrix effect (%) | | | Extraction recovery (%) | | |
| --- | --- | --- | --- | --- | --- | --- | --- |
|  |  | Theoreti-cal (%) | CV (%) | Bias (%) | Theoreti-cal (%) | CV (%) | Bias (%) |
| Total DTX | LQC (15) | 95.83 | 8.65 | −4.15 | 91.89 | 7.55 | 0.71 |
|  | HQC (3000) | 101.85 | 5.60 | 1.91 | 100.13 | 6.27 | 3.64 |
| Encapsulated DTX | LQC (100) | 103.08 | 7.45 | 6.94 | 99.39 | 5.47 | −2.34 |
|  | HQC (3000) | 111.19 | 13.4 | 2.42 | 99.61 | 5.62 | −2.15 |
| Free DTX | LQC (30) | 104.44 | 0.05 | −0.59 | 90.96 | 7.63 | 0.60 |
|  | HQC (3000) | 108.72 | 3.77 | 9.61 | 100.60 | 3.08 | −9.04 |

* DTX: docetaxel; CV: coefficient of variation; LQC: low quality control; HQC: high quality control.

Table S6 Stability of total, encapsulated, free DTX liposomes in rat plasma (n = 5).

| Analyte | Condition | | Concentration (ng·mL^−1^) | **Mean ± SD** | CV (%) | Bias (%) |
| --- | --- | --- | --- | --- | --- | --- |
| Total DTX | | 4°C stability for 12 h | LQC (15) | 16.3 ± 0.19 | 1.18 | 8.65 |
|  |  |  | MQC (300) | 332.6 ± 6.01 | 1.81 | 10.9 |
|  |  |  | HQC (3000) | 3347.52±35.3 | 1.05 | 11.6 |
|  |  | −80°C stability for 30 d | LQC (15) | 16.5 ± 0.90 | 5.43 | 10.1 |
|  |  |  | MQC (300) | 338.6 ± 2.70 | 0.80 | 12.9 |
|  |  |  | HQC (3000) | 3340.0±70.1 | 2.10 | 11.3 |
|  |  | 25°C stability for 12 h | LQC (15) | 16.4 ± 0.30 | 1.85 | 9.33 |
|  |  |  | MQC (300) | 334.4 ± 6.46 | 1.93 | 11.5 |
|  |  |  | HQC (3000) | 3222.9±92.9 | 2.88 | 7.43 |
| Encapsulated DTX | | 4°C stability for 12 h | LQC (100) | 115.0 ± 5.66 | 4.92 | 15.0 |
|  |  |  | MQC (300) | 315.0 ± 8.14 | 2.59 | 5.01 |
|  |  |  | HQC (3000) | 3121.5±79.4 | 2.54 | 4.05 |
|  |  | −80°C stability for 12 h | LQC (100) | 105.8 ± 5.08 | 4.80 | 5.79 |
|  |  |  | MQC (300) | 313.7 ± 8.74 | 2.78 | 4.55 |
|  |  |  | HQC (3000) | 3075.9±88.8 | 2.89 | 2.53 |
|  |  | 25°C stability for 12 h | LQC (100) | 93.9 ± 4.33 | 4.61 | −6.10 |
|  |  |  | MQC (300) | 289.3±13.82 | 4.78 | −3.58 |
|  |  |  | HQC (3000) | 3242.5±103.0 | 3.17 | 8.08 |
| Free DTX | | 4°C stability for 12 h | LQC (30) | 30.4 ± 0.98 | 3.23 | 1.48 |
|  |  |  | MQC (300) | 304.0 ± 12.8 | 4.21 | 1.32 |
|  |  |  | HQC (3000) | 3019.3 ± 83.1 | 2.75 | 0.64 |
|  |  | −80°C stability for 20 d | LQC (30) | 29.7 ± 1.08 | 3.63 | −1.13 |
|  |  |  | MQC (300) | 311.6 ± 7.34 | 2.36 | 3.87 |
|  |  |  | HQC (3000) | 3013.1± 112.3 | 3.73 | 0.43 |
|  |  | 25°C stability for 12 h | LQC (30) | 30.4 ± 0.82 | 2.71 | 1.35 |
|  |  |  | MQC (300) | 309.0 ± 5.96 | 1.93 | 3.00 |
|  |  |  | HQC (3000) | 2925.8± 107.1 | 3.66 | −2.47 |

* LQC: low quality control; MQC: medium quality control; HQC: high quality control.

Table S7 Pharmacokinetic Parameters of Intravenously Administered Docetaxel(DTX) Liposomes in Rats (n = 5 per Group): (a) Total DTX, (b) Encapsulated DTX, (c) Free DTX.

(a)

| Total DTX  PK parameters | Dose | | | |
| --- | --- | --- | --- | --- |
|  | 1 mg·kg^−1^ | 2 mg·kg^−1^ | | 4 mg·kg^−1^ |
| T_1/2_ (h) | 4.34 ± 0.43 | | 8.82 ± 2.94 | 8.33 ± 2.94 |
| C_max_ (ng·mL^−1^) | 1258.77 ± 351.38 | | 3803.28 ± 193.58 | 7184.01 ± 520.69 |
| AUC_0~t_ (ng·h·mL^−1^) | 1201.54 ± 122.03 | | 2800.00 ± 745.42 | 5951.08 ± 738.8 |
| AUC_0~∞_ (ng·h·mL^−1^) | 1451.27 ± 125.43 | | 3217.22 ± 792.92 | 6399.53 ± 701.93 |
| CL (mL·h^−1^·kg^−1^) | 697.84 ± 65.81 | | 655.23 ± 180.56 | 630.62 ± 63.74 |
| Vss (mL·kg^−1^) | 4301.11 ± 647.99 | | 5461.67 ± 1804.24 | 3919.75 ± 992.52 |

(b)

| Encapsulated DTX  PK parameters | Dose | | |
| --- | --- | --- | --- |
|  | 1 mg·mL^−1^ | 2 mg·mL^−1^ | 4 mg·mL^−1^ |
| T_1/2_ (h) | 6.35 ± 2.56 | 8.49 ± 6.57 | 11.86 ± 14.95^*^ |
| Cmax (ng·mL^−1^) | 1125.28 ± 322.46^a^ | 3150.45 ± 193.53^a^ | 6438.37 ± 324.92 |
| AUC_0~t_ (ng·h·mL^−1^) | 474.72 ± 159.65^a^ | 1004.47 ± 113.69^a^ | 2167.20 ± 865.50^a^ |
| AUC_0~∞_(ng·h·mL^−1^) | 924.77 ± 364.73^a^ | 1582.82 ± 517.98^a^ | 3408.71 ± 2363.79^a^ |
| CL (mL·h^−1^·kg^−1^) | 1241.49 ± 433.44 | 1365.15 ± 390.28 | 1564.59 ± 774.00 |
| V_ss_ (mL·kg^−1^) | 8446.08 ± 911.94 | 10615.37± 4948.69 | 10699.95± 8373.13 |

* At the highest dose (4 mg·kg⁻¹), the mononuclear phagocyte system (MPS) may become saturated, leading to more variable clearance kinetics among individual animals.

(c)

| Free DTX  PK parameters | Dose | | |
| --- | --- | --- | --- |
|  | 1 mg·mL^−1^ | 2 mg·mL^−1^ | 4 mg·mL^−1^ |
| T_1/2_ (h) | 3.90 ± 2.48 | 3.85 ± 1.16^b^ | 4.81 ± 0.75 |
| C_max_ (ng·mL^−1^) | 337.38 ± 19.96^b,c^ | 1668.33 ± 699.54^b,c^ | 2876.54 ± 252.24^b,c^ |
| AUC_0~t_ (ng·h·mL^−1^) | 555.65 ± 72.3^b^ | 1142.88 ± 335.84 ^b^ | 3195.21 ± 364.45^b^ |
| AUC_0~∞_(ng·h·mL^−1^) | 665.31 ± 123.06^b^ | 1236.09 ± 347.32^b,c^ | 3372.89 ± 311.07^b^ |
| CL (mL·h^−1^·kg^−1^) | 1550.96 ± 308.36 | 1758.75 ± 642.98 | 1194.05 ± 110.46 |
| V_ss_ (mL·kg^−1^) | 7806.79± 2318.68 | 8105.14 ± 3455.69 | 6179.04 ± 1114.26 |

a. Statistical significance was observed at *p* < 0.05 when comparing group 'a' to both the total and encapsulated DTX liposome groups at the same dose.
b. group 'b' demonstrated statistical significance at *p* < 0.05 when compared to the encapsulated and free DTX liposome groups at the same dose.
c. group 'c' showed statistical significance at *p* < 0.05 in comparison to the total and free DTX liposome groups at the same dose.

*	The experimental protocol was approved by the Animal Ethics Committee of the Beijing Institute of Pharmacology and Toxicology (Animal Certificate Number: SCXK (Jing) 2021–0006; Ethics Approval Number: IACUC-DWZX-2024-P693). All procedures involving animals complied with the Guidelines for the Care and Use of Laboratory Animals.
